# Editorial: The effect of gut microbiota on the brain structure and function

**DOI:** 10.3389/fnint.2023.1226664

**Published:** 2023-06-29

**Authors:** Shan Liang, Feng Jin, Chenxi Jia

**Affiliations:** ^1^Institute of Psychology, Chinese Academy of Sciences (CAS), Beijing, China; ^2^Institute of Microbiology, Chinese Academy of Sciences (CAS), Beijing, China; ^3^State Key Laboratory of Proteomics, National Center for Protein Sciences-Beijing, Beijing Proteome Research Center, Beijing Institute of Lifeomics, Beijing, China

**Keywords:** microbiota-gut-brain, gut microbiota, preterm birth, meningitis, lead, diet, Alzheimer's disease, Parkinson's disease

The theory of gut-brain axis become research hotspot since the decades, and the role of gut microbiota in mental disorders causes special attention. The proposition of gut-brain psychology in 2018 clearly stated the connection among gut, microbiota, brain and mental health (Liang et al., 2018). The Figure 1 shows the remarkable increasing number of articles in the PubMed.

**Figure 1:**
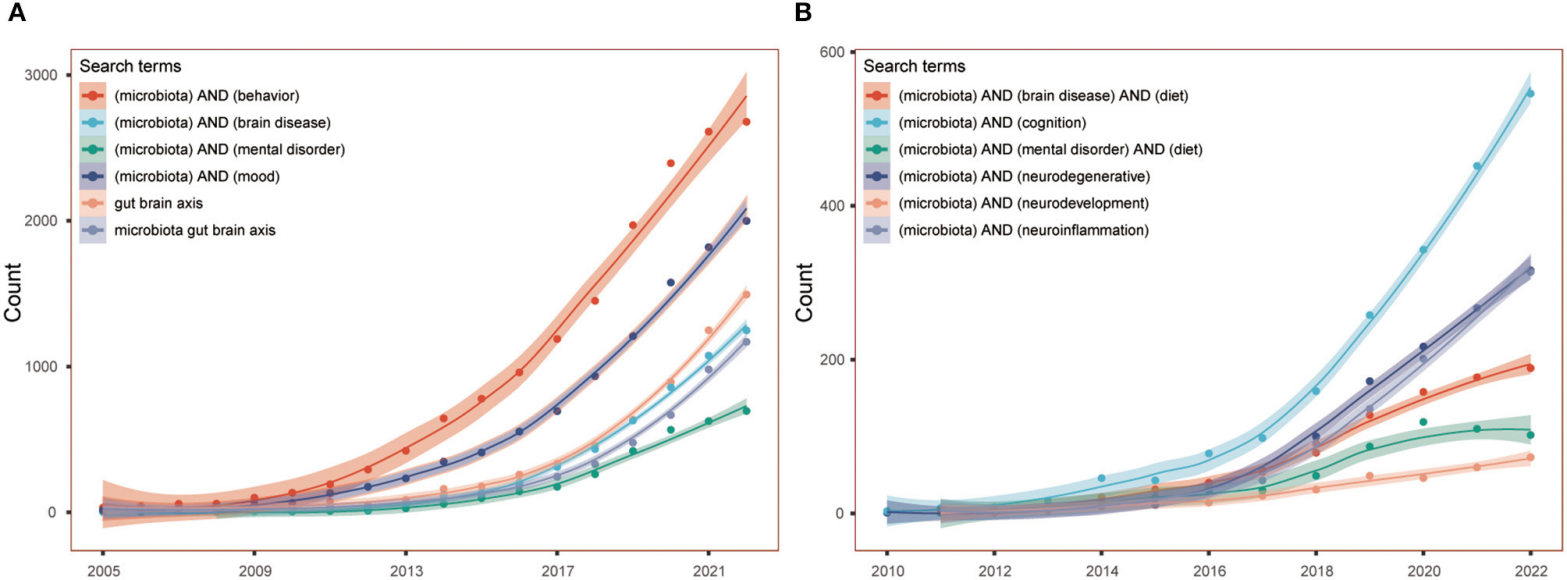
**(A, B)** The ever-increasing number of articles in PubMed in gut-brain axis field.

The change of gut microbiota and brain development almost synchronously occur throughout whole life. Disruption of the coordination may lead variety of brain diseases including neurodevelopmental and neurodegenerative diseases (Borre et al., 2014; Liang et al., 2018). Early development may influence an individual whole life. In this series, preterm birth as serious problem is concerned. When premature infants abnormally expose to unexpected environmental factors too early, they may have to face substantial risk of perinatal white matter injury (WMI) or other neurodevelopmental disorders. The study indicated that their gut microbiota and brain development are obviously impaired, the brain damage were correlated with the overgrowth of unfriendly bacteria like Klebsiella (Seki et al., 2021). Wang et al. summarized the role of microbiota-gut-brain axis under the WMI, pointed that the gut microbiota could influence premature brain through pathways including SCFAs production, cytokines regulation, and oxidative stress alleviation, and suggested probiotics and prebiotics therapy can be a promising way to improve WMI.

Gut microbiota also paly great role in preventing neonatal meningitis. Gut microbiota immaturity and epithelial barriers' permissiveness promote bacterial like Group B streptococcus dissemination from the gut to the brain and collectively account for neonatal susceptibility to bacterial meningitis (Travier et al., 2021). Gut-educated immune cells including IgA plasma cells, B cells, NK cells, and T cells, etc. could migrate to meninges to protect the brain parenchyma from pathogen infection. The meninges of germ-free rodents lacked these immune cells and normal gut microbiota might be the key factor of the migration (Fitzpatrick et al., 2020; Di Marco Barros et al., 2022). Administration of probiotics and/or prebiotics may help prevent neonatal bacterial meningitis by promoting the maturation of gut-meninges defense (Fitzpatrick et al., 2020; Travier et al., 2021; Di Marco Barros et al., 2022).

Diet is the most important factor in modulating gut microbiota, and it plays great role in the formation and function of microbiota-gut-brain axis after birth. Poor diet and harmful diet-related substances like heavy metal damage gut microbiota and brain health while healthy diet improves the both (Liang et al., 2018, 2022; Singh et al., 2022; Solch et al., 2022).

The effects of heavy metals on human health are well-known. Studies showed that heavy metal could impair microbiota-gut-brain axis (Lin et al., 2020; Sun et al., 2022; Yu et al., 2022) while probiotics and prebiotics alleviated the damage (Nagano et al., 2021; Yao et al., 2022; Zhang et al., 2023). The following study shows early lead exposure not only disturbed gut microbiota but also impaired mental health. Zhang et al. found lead exposure during lactation impaired gut microbiota and spatial memory of female offspring rats. Furthermore, their study showed that *Lacticaseibacillus rhamnosus* GR-1 supplementation alleviated the gut microbiota abnormalities and improved the memory deficits no matter before, during or after lead exposure. Although prophylactic gestational supplement did not decrease blood lead as postpartum supplement, it also regulated gut microbiota, produced the most prominent mitigation in spatial memory, and consumed lest time. Since the abundance of gut *Escherichia coli* was obviously negative correlated with spatial memory, researchers conducted further experiment. They found that *Lb. rhamnosus* supplement significantly improved *E. coli* O157 treatment induced memory deficits in rats with or without lead exposure. *Lactobacillus fermentum* HNU312 also improved gut microbiota and decreased anxiety-like and depression-like behavior in a chronic lead exposure mice model (Zhang et al., 2023). Both studies indicated the potential of certain probiotics in prevention and alleviation of heavy metal poisoning.

In addition to heavy metal, early life exposure to pesticides could disrupt microbiota-gut-brain axis development and induce autism spectrum disorders, attention deficit hyperactivity disorder (ADHD), tic disorders, and neurodegenerative disorders (Rude et al., 2019; Liang et al., 2022; Ramírez et al., 2022). Even the poor diet could interrupt microbiota-gut-brain axis and increase susceptibility to neuropsychiatric disorders during whole life through childhood to senile from autism, ADHD, Tourette syndrome, obsessive-compulsive disorder, depressive disorders, anxiety disorders, bipolar disorders, schizophrenia, to Alzheimer's disease, and Parkinson's disease (Liang et al., 2018, 2022; Solch et al., 2022).

The role of healthy diet and beneficial diet-related factors in preventing gut microbiota dysbiosis and brain diseases also received increasing attention (Liang et al., 2022; Solch et al., 2022). Franzoni et al. highlighted the role of antioxidant properties of several dietary compounds including Vitamin C and E, omega-3 polyunsaturated fatty acids, coenzyme Q10, chlorogenic acid, selenium, and probiotics in neuroprotection, and emphasized that these dietary combinations could be indispensable in prevention of neurodegenerative diseases.

Alzheimer's disease (AD) becomes more and more incidence in recent years (Liang et al., 2022). And increasing research indicate the effects of abnormal gut microbiota and poor diet in AD (Solch et al., 2022; Trejo-Castro et al., 2022). Bello-Corral et al. presented that the characteristic gut microbiota composition in AD patients was linked to the formation of beta-amyloid peptides, increase in systematic inflammation, and subsequent decline in cognition. The article also underlined the role of diet in AD. Healthy eating including Mediterranean diet and Japanese diet rich in omega-3 polyunsaturated fatty acids and antioxidant substances could help in AD prevention, while poor diet and processed foods would increase the risk of AD.

Parkinson's disease (PD) is another neurodegenerative disease keeping on a rise in the last decades (Liang et al., 2022). The association between PD and gut microbiota and diet has attracted more and more attention recently (Solch et al., 2022). The misfolding, aggregation and deposition of α-synuclein (α-syn) are major characteristics of PD. But the gut microbiota dysbiosis is an important risk factor of the development of α-syn (Nielsen et al., 2021), while polyphenols with great antioxidant properties help not only gut microbiota regulation but also neuroprotection. Although the positive effect of polyphenols in preventing neurodegenerative diseases has been focused, the mechanisms are still unclear (Zhang et al., 2022). In the present study, Yamasaki et al. provided some interesting clues, they found that phenolic acid compounds modified by gut microbiota not only inhibited the seeding aggregation of α-syn within biosensor cells, but also inhibited generation of aggregate-prone forms for both multiple system atrophy and PD “low seeder” brain samples. This positive observation merits special attention.

Huge researches have also presented the potential of probiotics, prebiotics, healthy diet, and antioxidant substances like polyphenols in regulating microbiota-gut-brain axis and improving brain disorders including autism spectrum disorders, Down's syndrome, ADHD, epilepsy, depressive disorders, anxiety disorders, and schizophrenia (Liang et al., 2018, 2022; Serra et al., 2020; Horn et al., 2022).

Overall, the present Research Topic focused on the association between gut microbiota and brain development and neurodegeneration, introduced some potential protective effects of dietary compounds like polyphenols in neurodegenerative disorders, showed the impairment of early lead exposure in cognition, and hinted the potential of probiotics intervention in these disorders. More fascinating studies are expected in the future.

## Author contributions

All authors listed have made a substantial, direct, and intellectual contribution to the work and approved it for publication.
